# Predictive value of cervical length for spontaneous preterm birth in women with cervical cerclage

**DOI:** 10.1002/uog.29281

**Published:** 2025-07-09

**Authors:** K. E. Mountain, S. Ng, T. Elger, H. Judah, R. Akolekar, H. V. Lewis, D. A. MacIntyre, V. Terzidou, P. R. Bennett, T. G. Teoh, L. Sykes, K. H. Nicolaides

**Affiliations:** ^1^ March of Dimes Prematurity Research Centre at The Institute of Reproductive and Developmental Biology, Department of Metabolism Digestion and Reproduction, Imperial College London London UK; ^2^ The Parasol Foundation Centre for Women's Health and Cancer Research Imperial College Healthcare NHS Trust London UK; ^3^ King's College Hospital NHS Foundation Trust London UK; ^4^ Medway NHS Foundation Trust Gillingham UK; ^5^ Chelsea and Westminster NHS Foundation Trust London UK; ^6^ Queen Charlotte's and Chelsea Hospital Imperial College Healthcare NHS Trust London UK; ^7^ St Mary's Hospital, Imperial College Healthcare NHS Trust London UK; ^8^ Harris Birthright Research Centre for Fetal Medicine, King's College London London UK

**Keywords:** cervical cerclage, cervical length, perinatology, spontaneous preterm birth

## Abstract

**Objective:**

Cervical cerclage is an important treatment used to prevent spontaneous preterm birth (sPTB), but it is not universally successful. Understanding the factors associated with cerclage treatment failure may lead to improved patient selection and better patient outcomes. The objective of this study was to investigate the value of pre‐ and post‐cerclage cervical length (CL) in predicting sPTB < 34 weeks.

**Methods:**

This was a retrospective cohort study conducted in four preterm birth prevention clinics in the UK. We included 331 women who had undergone cervical cerclage between January 2008 and March 2021, and analyzed their pre‐ and post‐cerclage CL, as measured by transvaginal ultrasound scan. The primary outcome was sPTB < 34 weeks' gestation, assessed using multivariable logistic regression modeling (variables were pre‐ and post‐cerclage CL, gestational age at cerclage and direction of CL change) and the generation of receiver‐operating‐characteristic (ROC) curves. Differences in sPTB for underlying risk factors (race, smoking history, previous cervical surgery or pregnancy history risk factors, including mid‐trimester loss or sPTB) were assessed using Fisher's exact test.

**Results:**

Both pre‐ and post‐cerclage CL were independently discriminative of sPTB < 34 weeks' gestation, with areas under the ROC curve of 0.635 (95% CI, 0.559–0.712) and 0.677 (95% CI, 0.604–0.751), respectively, and were modest contributors to sPTB prediction based on multivariable logistic regression modeling (odds ratio (OR), 0.964 (95% CI, 0.936–0.994), *P* = 0.018 and 0.940 (95% CI, 0.910–0.970), *P* < 0.001, respectively). There were no significant differences in the rate of sPTB < 34 weeks' gestation according to race, smoking history, previous cervical surgery or pregnancy history risk factors, including previous mid‐trimester loss or sPTB.

**Conclusions:**

Post‐cerclage CL is the predominant predictor of sPTB < 34 weeks' gestation. Underlying sPTB risk factors (previous cervical surgery and pregnancy history) may influence pre‐cerclage CL and the direction of CL change following cerclage, but once these are adjusted for, they do not influence the risk of sPTB < 34 weeks' gestation. © 2025 The Author(s). *Ultrasound in Obstetrics & Gynecology* published by John Wiley & Sons Ltd on behalf of International Society of Ultrasound in Obstetrics and Gynecology.

## INTRODUCTION

Preterm birth (PTB) is the leading cause of neonatal morbidity and mortality worldwide^1^. The majority of PTBs are termed spontaneous (preterm labor or preterm prelabor rupture of membranes (PPROM))^2^. Cervical cerclage, a procedure in which a suture is used to provide mechanical support to the cervix, is a common intervention for spontaneous PTB (sPTB) prevention^3^. The Royal College of Obstetricians and Gynaecologists recommend offering cervical cerclage to women with a history suggestive of cervical insufficiency (i.e. multiple previous sPTBs) and those with recognized sPTB risk factors who have a short cervical length (CL) (≤ 25 mm)^4^. Additionally, in those with an incidental finding of open cervix in the mid‐trimester, emergency cervical cerclage may be considered.

Cervical cerclage and alternative interventions, such as progesterone, are not universally successful in preventing PTB, irrespective of the indication for treatment^3^, ^5^, ^6^, ^7^, ^8^, ^9^, ^10^. Understanding the factors associated with intervention failure can aid patient selection for treatment with cerclage. Assessment of the cervix via transvaginal ultrasound (TVS) is the predominant screening method for pregnant women at risk of sPTB, as short CL is the strongest predictor of sPTB^11^, ^12^, ^13^. The value of TVS after cervical cerclage insertion is unclear. Several studies^14^, ^15^, ^16^, ^17^, ^18^, ^19^, ^20^ have attempted to define predictive parameters for cerclage failure using TVS but the results have been variable, possibly due to small sample sizes and heterogeneity in study populations. In women with a history of cervical surgery, PTB may be mechanical in etiology, as the risk of PTB is correlated with cone depth^21^. In women with a history of PTB or mid‐trimester loss (MTL), a microbial/inflammatory etiology is more often observed^22^. Therefore, response to cervical cerclage and changes in CL may differ between these women.

The primary study objective was to evaluate the predictive value of CL screening, both before and after insertion of cervical cerclage, for sPTB < 34 weeks' gestation in a large population of at‐risk women, accounting for gestational age at cerclage and risk factors for sPTB.

## METHODS

### Study design, setting and population

This was a retrospective cohort study of women who received CL screening and underwent cervical cerclage during pregnancy at one of four PTB prevention clinics in the UK (Imperial College Healthcare NHS Trust, London; Chelsea and Westminster Hospital, London; King's College NHS Foundation Trust, London; and Medway NHS Foundation Trust, Kent) between January 2008 and March 2021. Ethical approval was not sought or required as the study involved retrospective analysis of routinely collected non‐identifiable clinical data.

Women who underwent cervical cerclage in pregnancy were included if they had serial CL measurements taken during the pregnancy, with at least one measurement available for both before and after insertion of cervical cerclage. Women who received progesterone therapy in conjunction with cervical cerclage were excluded. Women were also excluded when their risk profile for PTB could not be determined or when data on maternal race or smoking history were missing. Furthermore, women were excluded when gestational age at delivery or onset of labor were uncertain. Routinely collected data included maternal age, body mass index (BMI), race, smoking history, risk factors for sPTB (prior history of cervical surgery, MTL, sPTB and cervical cerclage), CL, indication for cervical cerclage, gestational age at insertion of cervical cerclage, use of progesterone, onset of labor and gestational age at delivery. Data were gathered from previously collected audit data (at all sites, including Chelsea and Westminster Hospital) as well as electronic patient records and scanning systems (Cerner (EPR Solutions, North Kansas City, MO, USA) at Imperial College Healthcare; Viewpoint version 5.6 (GE Healthcare, Buckinghamshire, UK) at Kings College Hospital and Medway Maritime Hospital).

CL was measured as recommended by the Cervical Length Education and Review program (Perinatal Quality Foundation, OK, USA)^23^, and as described by Mella and Berghella^24^. In brief, women were scanned with an empty bladder and the probe was inserted into the anterior fornix of the vagina, obtaining a sagittal view along the cervical canal. Care was taken not to elongate the cervix by exerting excessive pressure, and CL was measured from internal to external os. All operators were trained in this technique. A cervix was considered short when the total CL was ≤ 25 mm. The decision to insert cervical cerclage was based on clinical history, TVS findings or both.

### Outcomes and covariables

The primary outcome was sPTB < 34 weeks' gestation. PTB was considered spontaneous when onset of labor was by cervical dilatation or PPROM. Women who had medically indicated PTB were excluded. Women who had PTB between 34 + 0 and 36 + 6 weeks' gestation were not considered since delivery < 34 weeks has been more closely associated with cervical change and adverse neonatal outcomes^11^, ^25^.

CL is known to decrease with gestational age^26^ and it has been suggested that this may affect its predictive value^27^. To account for different gestational ages at CL measurement and cerclage insertion, the gestational age at each of these timepoints was included as a variable in the modeling analyses.

### Statistical analysis

All statistical analyses were performed using R (v4.0.0)^28^. All data were visualized using the ‘ggplot2’ package (v3.3.5)^29^, unless stated otherwise. Women who had missing data for age (*n* = 3) and BMI (*n* = 53) had values imputed based on the median value corresponding to their delivery‐outcome group (sPTB < 34 weeks or term delivery ≥ 37 weeks) using the ‘Hmisc’ package (v4.6.0)^30^. ROC curves and area under the ROC curve (AUC) values were generated using the ‘pROC’ package (v1.18.0)^31^. The direction for ROC comparison was set to ‘>’ to denote that samples in the control group (term delivery ≥ 37 weeks) had a higher CL compared to those in the case group (sPTB < 34 weeks). The DeLong method^32^ was used to compare AUC values between ROC curves. The Shapiro–Wilk test was used to determine normality for continuous variables, with *P* > 0.05 indicative of normally distributed data. The Mann–Whitney *U*‐test was used to assess statistical differences for all continuous variable comparisons to ensure consistency in the method used to compare overall distributions between groups and due to this test being less sensitive to outliers. All categorical variables had cells with frequency ≤ 5, except for the ‘previous cervical surgery’ and ‘indication for cerclage’ variables; therefore, the Fisher's exact test was used to determine associations for all categorical variables. A *P*‐value < 0.05 was used to determine statistical significance for all statistical tests.

The value of CL measurement pre‐ and post‐cerclage for predicting sPTB < 34 weeks was assessed using multivariable logistic regression with backward selection using the ‘caret’ package (v6.0.90)^33^, with gestational age at delivery (weeks) as the binary outcome (sPTB < 34 weeks or term delivery ≥ 37 weeks). Independent variables included pre‐cerclage CL, post‐cerclage CL, gestational age at cerclage and direction of CL change, which was classified as an increase (> 2 mm increase between pre‐ and post‐cerclage measurements), no change (≤ 2 mm change in either direction) or a decrease (> 2 mm decrease between pre‐ and post‐cerclage measurements) in CL, following one‐hot encoding. The variance inflation factor (VIF) was used to assess collinearity between variables, with VIF > 5 indicating collinearity. All variables included in logistic regression models had VIF < 5.

## RESULTS

### Participant characteristics

A total of 5572 patient records were identified for potential inclusion, of which 1145 underwent cerclage during pregnancy; 524 of these were excluded due to concurrent use of progesterone. Of the remaining 621 patients, 432 patients had both pre‐ and post‐cerclage scans. Of these, 70 were excluded due to delivery between 34 + 0 and 36 + 6 weeks and 31 were excluded due to significant missing data, leaving a final cohort of 331 patients (Figure S1). The median gestational age was 15 (interquartile range (IQR), 14–19) weeks at the pre‐cerclage scan, 20 (IQR, 18–23) weeks at the post‐cerclage scan and 17 (IQR, 15–20) weeks at cerclage insertion. The median interval between the pre‐cerclage scan and cerclage insertion was 4 (IQR, 2–5) days and that between cerclage insertion and the post‐cerclage scan was 16 (IQR, 8–28) days.

### Outcome and cervical length according to risk factors

There were no significant differences in maternal race, smoking history, pregnancy history or previous cervical surgery between women who had sPTB < 34 weeks and those who delivered at term. While there was a statistically significant difference between the two groups in terms of median maternal age (33 (IQR, 30–37) years in the term‐delivery cohort *vs* 32 (IQR, 28–35) years in the sPTB cohort), the absolute difference was small and not clinically relevant. There was evidence of an association between indication for cerclage and sPTB, with patients who had history‐indicated cerclage being less likely to experience sPTB. Specifically, 11.9% of those with history‐indicated cerclage experienced sPTB, compared to 24.3% with ultrasound‐indicated cerclage and 36.8% with emergency cerclage (*P* = 0.003) (Table 1).

**Table 1:** Demographic characteristics of 331 women who underwent cervical cerclage, according to whether they had spontaneous preterm birth (sPTB) < 34 weeks' gestation or delivered at term (≥ 37 weeks' gestation)

Parameter	sPTB (*n* = 65)	Term delivery (*n* = 266)	*P*
Age (years)	32 (28–35)	33 (30–37)	0.039
BMI (kg/m^2^)	25 (23–30)	27 (23–30)	0.088
Gestational age at delivery (weeks)	27 (23–30)	39 (38–40)	< 0.001
Race			0.210
Asian	1 (1.5)	18 (6.8)	
Black	32 (49.2)	112 (42.1)	
Mixed	1 (1.5)	13 (4.9)	
Other	4 (6.2)	8 (3.0)	
White	27 (41.5)	115 (43.2)	
Pregnancy history			0.320
Nulliparous	16 (24.6)	47 (17.7)	
Nulliparous with previous MTL	15 (23.1)	45 (16.9)	
Parous with previous MTL	10 (15.4)	53 (19.9)	
Parous with previous iPTB	1 (1.5)	1 (0.4)	
Parous with previous sPTB	18 (27.7)	92 (34.6)	
Parous with previous term delivery only	5 (7.7)	28 (10.5)	
Smoking history	5 (7.7)	11 (4.1)	0.330
Previous cervical surgery	17 (26.2)	63 (23.7)	0.750
Indication for cervical cerclage			0.003
Emergency	7 (10.8)	12 (4.5)	
History‐indicated	17 (26.2)	126 (47.4)	
Ultrasound‐indicated	41 (63.1)	128 (48.1)	

Data are given as median (interquartile range) or *n* (%).

BMI, body mass index; iPTB, iatrogenic/indicated preterm birth; MTL, mid‐trimester loss.

In women who had undergone previous cervical surgery, pre‐cerclage CL measurements were significantly lower compared to those in women without a history of cervical surgery (*P* = 0.0002) (Table S1). There was no significant difference in post‐cerclage CL between women with and those without a history of cervical surgery. Nulliparous women and women with only previous term delivery had significantly shorter pre‐cerclage CL than those with other pregnancy history (Figure S2). Women who had previous MTL and women who had previous sPTB had similar pre‐cerclage CL (*P* = 0.722). White women had significantly shorter pre‐cerclage CL compared with Black women (*P* = 0.001) or those of ‘other’ race (*P* = 0.046). The difference in CL between White and Black women persisted after cerclage insertion (*P* = 0.003), and in addition, White women had significantly shorter post‐cerclage CL compared with Asian women (*P* = 0.046).

### Change in cervical length according to risk factors

Women in whom CL decreased after cervical cerclage had a higher pre‐cerclage CL compared to women in whom CL increased (*P* < 0.001). Significantly more women who had undergone previous cervical surgery experienced an increase in CL after cerclage compared with those who had not (*P* = 0.001) (Table 2). Similarly, there were significant differences in the direction of CL change after cerclage according to pregnancy history (*P* = 0.001). A greater proportion of nulliparous women and parous women with previous term delivery experienced an increase in CL post cerclage compared to parous women with previous PTB and those with previous MTL. A significantly greater proportion of nulliparous women who had previous MTL experienced a decrease in CL after cerclage, and a greater proportion of parous women who had previous MTL had an unchanged CL post cerclage compared to those with other pregnancy history, with the exception of parous women with previous iatrogenic/indicated PTB. There were no significant differences in the direction of CL change according to smoking history or race (Table 2).

**Table 2:** Proportion of women who had increase, decrease and no change in cervical length after cerclage insertion, according to risk factor

	Cervical length			
Parameter	Decrease	Increase	No change	*P*
Previous cervical surgery				0.001
No	70 (27.9)	115 (45.8)	66 (26.3)	
Yes	8 (10.0)	52 (65.0)	20 (25.0)	
Pregnancy history				0.001
Nulliparous	5 (7.9)	41 (65.1)	17 (27.0)	
Nulliparous with previous MTL	24 (40.0)	23 (38.3)	13 (21.7)	
Parous with previous MTL	18 (28.6)	22 (34.9)	23 (36.5)	
Parous with previous iPTB	0 (0)	1 (50.0)	1 (50.0)	
Parous with previous sPTB	26 (23.6)	58 (52.7)	26 (23.6)	
Parous with previous term delivery only	5 (15.2)	22 (66.7)	6 (18.2)	
History of smoking				0.170
No	73 (23.2)	157 (49.8)	85 (27.0)	
Yes	5 (31.2)	10 (62.5)	1 (6.2)	
Race				0.370
Asian	3 (15.8)	12 (63.2)	4 (21.0)	
Black	35 (24.3)	70 (48.6)	39 (27.1)	
Mixed	3 (21.4)	4 (28.6)	7 (50.0)	
Other	5 (41.7)	4 (33.3)	3 (25.0)	
White	32 (22.5)	77 (54.2)	33 (23.2)	

Data are given as *n* (%).

iPTB, iatrogenic/indicated preterm birth; MTL, mid‐trimester loss; sPTB, spontaneous preterm birth.

### Predictive value of cervical length measurements pre‐ and post‐cerclage

**Figure 1:**
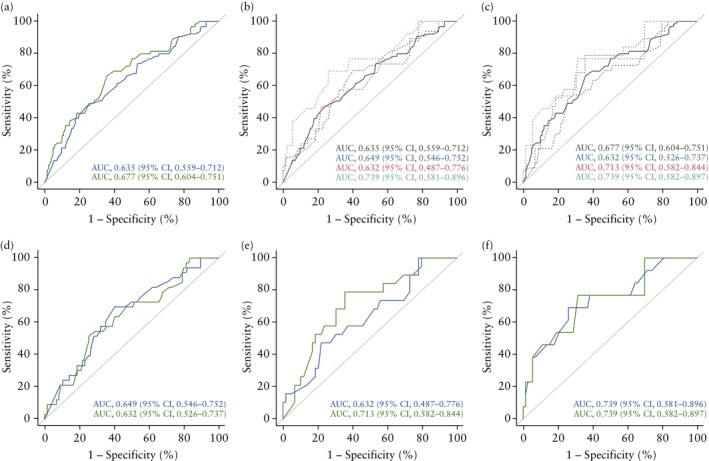
Receiver‐operating‐characteristics curves for prediction of spontaneous preterm birth < 34 weeks based on cervical length (CL). (a) Comparison between pre‐cerclage CL (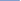) and post‐cerclage CL (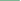) in all women. (b,c) Pre‐cerclage (b) and post‐cerclage (c) CL in all women (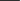) compared with those who had an increase (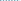), decrease (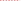) and no change (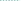) in CL after cerclage. (d–f) Comparison between pre‐cerclage CL (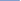) and post‐cerclage CL (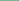) in women who had increase (d), decrease (e) or no change (f) in CL after cerclage. Areas under the receiver‐operating‐characteristics curves (AUCs) are shown.

Both pre‐ and post‐cerclage CL were discriminative of sPTB < 34 weeks. For the pre‐cerclage measurement, the AUC was 0.635 (95% CI, 0.559–0.712) (Figure 1), with 75.6% (95% CI, 70.1%–80.3%) specificity, 47.7% (95% CI, 36.0%–59.6%) sensitivity, 85.5% (95% CI, 80.5%–89.5%) negative predictive value (NPV) and 32.3% (95% CI, 23.8%–42.2%) positive predictive value (PPV) at an optimum threshold of 20.2 mm (Figure S3), which was based on the cut‐off with the highest Youden index. For the post‐cerclage measurement, the AUC was 0.677 (95% CI, 0.604–0.751), with 64.3% (95% CI, 58.4%–69.8%) specificity, 66.2% (95% CI, 54.0%–76.5%) sensitivity, 88.6% (95% CI, 83.3%–92.4%) NPV and 31.2% (95% CI, 24.0%–39.3%) PPV at an optimum threshold of 25.7 mm. Comparison of the ROC curves showed no significant differences between pre‐ and post‐cerclage CL measurements (Figure 1a). The ROC curves for pre‐ and post‐cerclage CL measurements were largely unchanged when compared across the categories of direction of CL change (Figure 1b,c). Similarly, there were no significant differences between the ROC curves for pre‐ and post‐cerclage CL measurements when stratified according to direction of CL change (Figure 1d–f).

### Predictive models for spontaneous preterm birth < 34 weeks

Initial modeling, including gestational age at cerclage, indication for cerclage, race, pregnancy history, previous cervical surgery and direction of CL change, as well as the intervals between pre‐cerclage CL measurement, cerclage insertion and post‐cerclage CL measurement, along with pre‐cerclage and post‐cerclage CL, demonstrated that only pre‐ and post‐cerclage CL were potentially significant for the prediction of sPTB < 34 weeks. A model including gestational age at cerclage, due to the known association between gestational age and CL, and pre‐ and post‐cerclage CL measurements demonstrated that both pre‐cerclage CL (odds ratio (OR), 0.964 (95% CI, 0.936–0.994); *P* = 0.018) and post‐cerclage CL (OR, 0.940 (95% CI, 0.910–0.970); *P* < 0.001) were significant predictors for sPTB < 34 weeks (Table S2). A final model comprising pre‐ and post‐cerclage CL only demonstrated that the effect of post‐cerclage CL on sPTB < 34 weeks was greater than that of pre‐cerclage CL (OR, 0.943 (95% CI, 0.914–0.973); *P* < 0.001 *vs* OR, 0.974 (95% CI, 0.949–1.001); *P* = 0.058) (Table S3).

## DISCUSSION

CL prior to and after insertion of cervical cerclage is predictive of sPTB < 34 weeks. In our model, the post‐cerclage measurement correlated more strongly with sPTB < 34 weeks than the pre‐cerclage measurement. Neither the direction of CL change nor underlying risk factors for sPTB influenced the predictive value of pre‐ or post‐cerclage CL. However, this study demonstrates that women with different demographics and risk factors for sPTB have pre‐existing differences in CL before cerclage. These differences are largely diminished once the cerclage is inserted. In turn, this means that women with different risk factors for sPTB are more likely to experience certain directions of CL change; women who have undergone cervical surgery have shorter pre‐cerclage CL and a greater proportion of these women experience an increase in post‐cerclage CL compared to those in the population who have not undergone cervical surgery. Women with pregnancy history associated with sPTB risk (previous MTL or sPTB) had longer pre‐cerclage CL but a smaller proportion had an increase in post‐cerclage CL, with a greater proportion of parous women with previous MTL having unchanged CL and more nulliparous women with previous MTL having decreased CL after cerclage, compared to women with other pregnancy history.

In agreement with previous studies, our findings suggest that post‐cerclage CL is an important predictor of sPTB^14^, ^17^, ^18^, ^19^. However, unlike studies confined to only women who have had previous MTL or sPTB^19^, or to particular indications for cerclage (e.g. excluding history‐indicated cerclage)^17^, our data include and account for women with a range of risk factors for sPTB and women with different indications for cerclage. Unlike previous studies^14^, ^20^, we found no impact of the direction of CL change on the risk of sPTB. However, these studies did not account for underlying differences in CL in these groups. Our findings suggest that previous work highlighting the importance of a change in CL after cerclage is confounded by collinearity with total CL, both pre‐ and post‐cerclage, and therefore, total CL is the most robust predictor of sPTB post cerclage.

The difference in pre‐cerclage CL and that in the direction of CL change after cerclage according to sPTB risk factors is a novel finding. It is possible that, in women with a history of MTL or PTB, the threshold for clinician decision‐making regarding cerclage insertion typically occurs at a longer CL than in women who have had cervical surgery. However, this would not account for the differences in the proportion of women experiencing a change in CL post cerclage. It is plausible that this relates to different underlying mechanisms for CL change in these groups, which may respond differently to cerclage insertion. For example, in those who have had cervical surgery, mechanical weakness may result in cervical shortening^21^, which can be reversed with the insertion of cerclage. By comparison, in those with a history of MTL, host–microbial interactions and early cervical remodeling could be responsible for cervical shortening, which are processes that might not be reversible and might be dependent on cerclage technique or material^22^, ^34^, ^35^, ^36^. Exploring these differences further may allow us to maximize the potential benefit women receive from undergoing cerclage insertion by maximizing their chances of a longer post‐cerclage CL.

The value of post‐cerclage CL in predicting subsequent sPTB < 34 weeks demonstrates the importance of ongoing CL surveillance following cerclage. Furthermore, it reinforces earlier studies that suggest that surgical techniques which maximize cerclage height may prolong gestation and supports the need to achieve the longest possible CL with cerclage placement^14^, ^15^.

The main limitation of this study is that the cohort includes only women who have undergone cervical cerclage; therefore, the optimal CL threshold generated prior to cerclage cannot be used to aid or modify decision‐making. However, this was not the purpose of reporting the threshold; rather, the threshold was used mainly to determine the optimal AUC achievable from pre‐cerclage CL. This study has also not assessed any metrics relating to cerclage position within the cervical canal. However, as discussed above, post‐cerclage CL is a good predictor of sPTB and this is likely to be associated closely with cerclage position. To ensure all analyses could be performed in the same population, women with missing age or BMI data had median imputation performed according to their delivery‐outcome group and women were excluded if they had missing race or smoking history data, both of which could potentially introduce statistical or sampling bias that influences model performance. The intervals between pre‐ and post‐cerclage scans and insertion of cervical cerclage were not standardized. However, logistic regression analysis showed that this did not influence our findings.

This study has several strengths, including its large sample size, covering one of the most common risk factors for PTB and involvement of multiple institutions. These factors limit the risk of confounding according to underlying sPTB risk factors or surgical techniques, thereby making the findings more widely applicable.

In conclusion, when CL is accounted for, the risk for sPTB < 34 weeks across sPTB risk factors is consistent. However, underlying risk factors for sPTB, including pregnancy history and previous cervical surgery, may influence the pre‐cerclage CL and direction of CL change following cerclage. Post‐cerclage CL is the predominant predictor of cerclage success for preventing sPTB, irrespective of the indication for cerclage; therefore, clinicians performing cervical cerclage should aim to maximize total CL when inserting cerclage and continue to monitor CL after cerclage insertion. Further research exploring the mechanisms behind the differences in pre‐ and post‐cerclage CL according to risk factors for sPTB may allow us to maximize the potential benefit women receive from undergoing cerclage insertion by maximizing their chances of having a longer post‐cerclage CL.

## Supporting information


**Figure S1** Flowchart showing study design.

**Figure S2** Box‐and‐whiskers plots showing pre‐ and post‐cerclage cervical length according to pregnancy history and race. Boxes show median and interquartile range and whiskers are range.

**Figure S3** Optimal threshold for pre‐ and post‐cerclage cervical length.

**Table S1** Pre‐ and post‐cerclage cervical length (CL) across risk factor groups
**Table S2** Logistic regression analysis to determine contribution of pre‐ and post‐cerclage length (CL) and gestational age at cerclage on delivery outcome
**Table S3** Logistic regression analysis to determine contribution of only pre‐ and post‐cerclage cervical length (CL) on delivery outcome

## Data Availability

The data that support the findings of this study are available from the corresponding author upon reasonable request.
