# Supplementary material for: Predictive value of cervical length for spontaneous preterm birth in women with cervical cerclage
Source: Ultrasound Obstet Gynecol. 2025 Jul 9;66(2):210–6. doi: 10.1002/uog.29281 (PMC12317304; doi:10.1002/uog.29281)
Supplement: Supplementary file 1 — Figure S1 Flowchart showing study design. [file UOG-66-210-s002.pptx]

## Slide 1
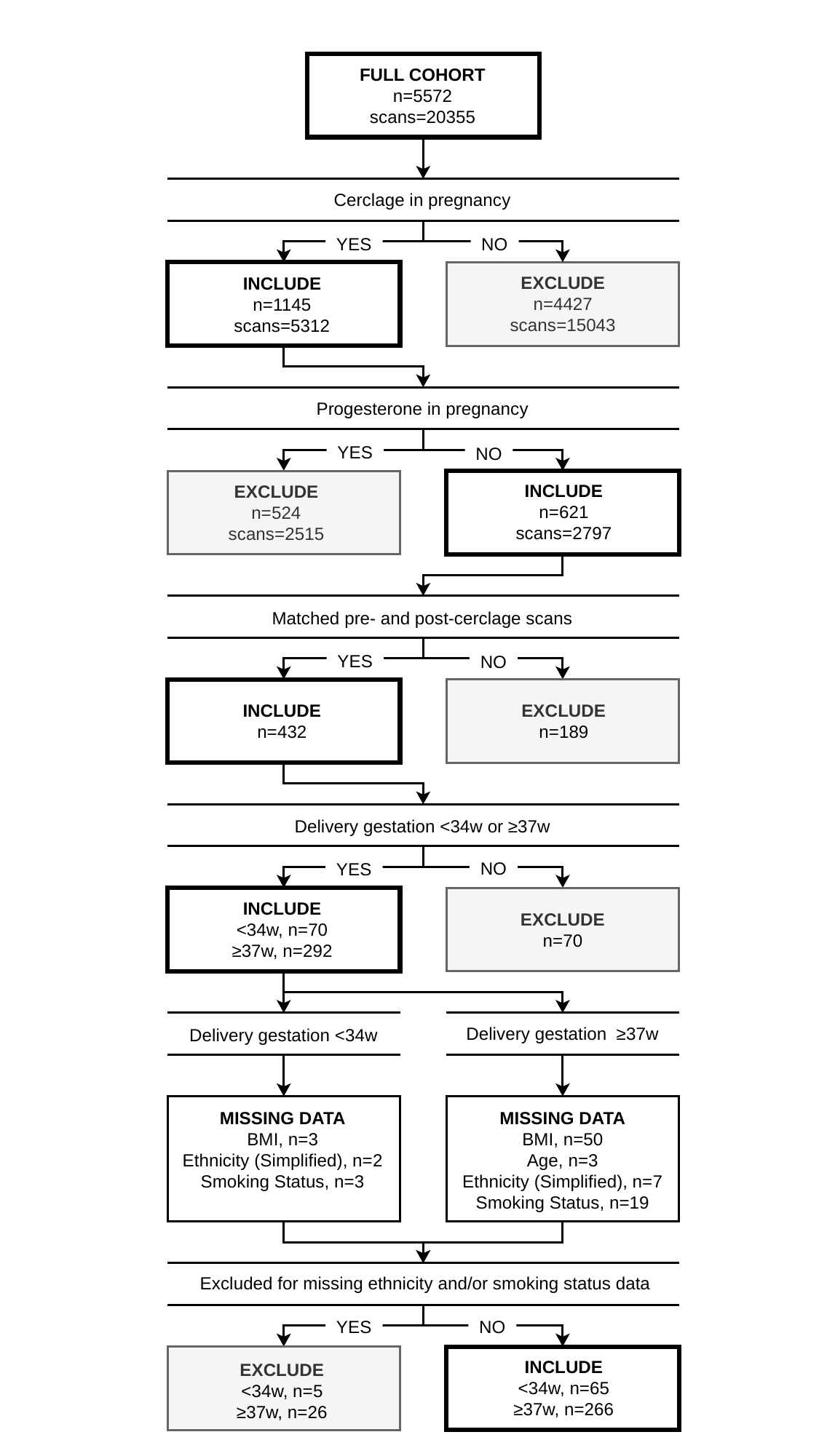

FULL COHORT
n=5572
scans=20355
Cerclage in pregnancy
YES
NO
EXCLUDE
n=4427
scans=15043
INCLUDE
n=1145
scans=5312
Progesterone in pregnancy
YES
NO
INCLUDE
n=621
scans=2797
EXCLUDE
n=524
scans=2515
Matched pre- and post-cerclage scans
YES
NO
INCLUDE
n=432
EXCLUDE
n=189
Delivery gestation <34w or ≥37w
NO
YES
INCLUDE
<34w, n=70
≥37w, n=292
EXCLUDE
n=70
Delivery gestation  ≥37w
Delivery gestation <34w
MISSING DATA
BMI, n=3
Ethnicity (Simplified), n=2
Smoking Status, n=3
Excluded for missing ethnicity and/or smoking status data
YES
NO
INCLUDE
<34w, n=65
≥37w, n=266
EXCLUDE
<34w, n=5
≥37w, n=26
MISSING DATA
BMI, n=50
Age, n=3
Ethnicity (Simplified), n=7
Smoking Status, n=19
