# Supplementary figures and images for: Predictive value of cervical length for spontaneous preterm birth in women with cervical cerclage
Source: Ultrasound Obstet Gynecol. 2025 Jul 9;66(2):210–6. doi: 10.1002/uog.29281 (PMC12317304; doi:10.1002/uog.29281)

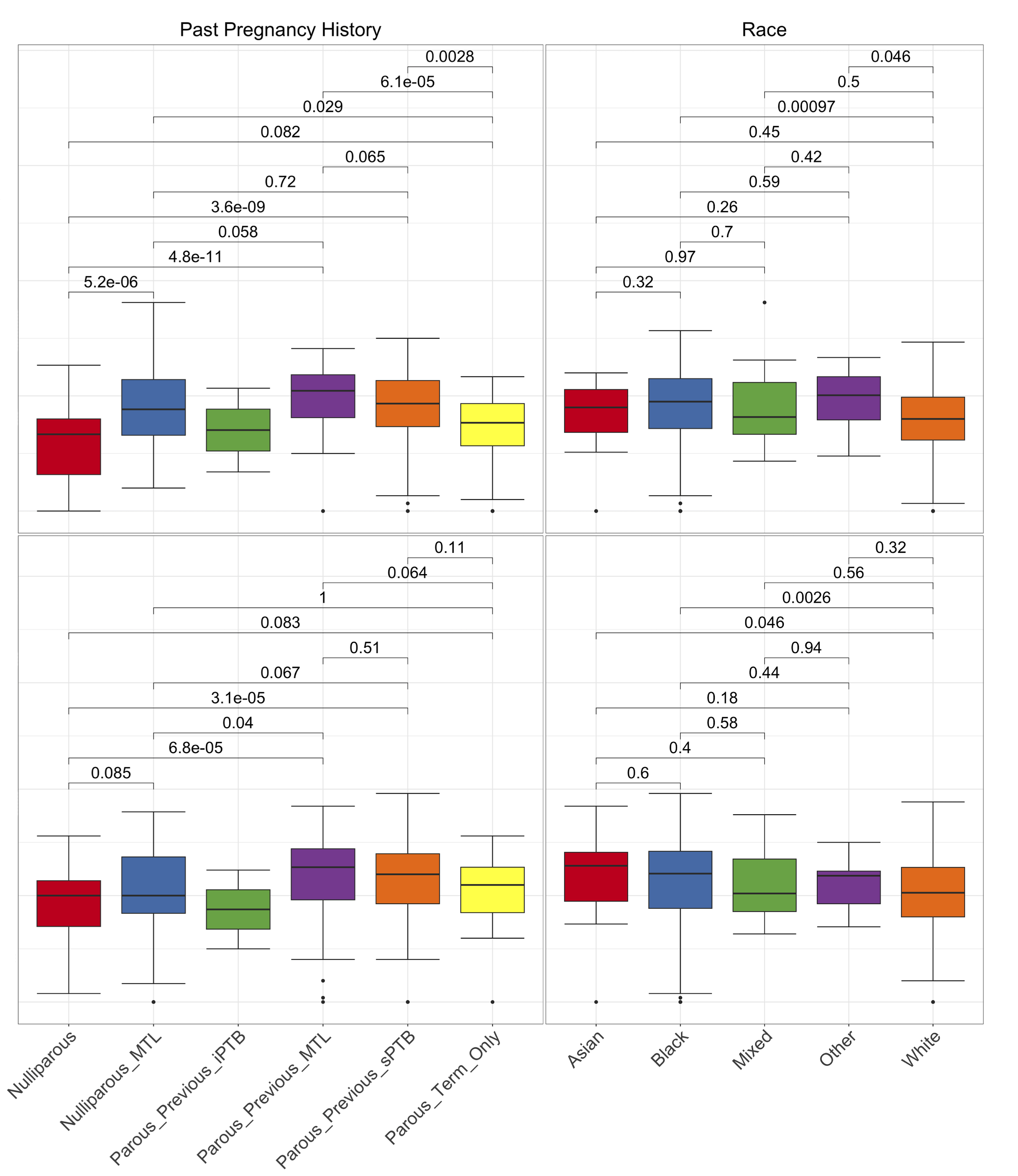

Supplement: Supplementary file 2 — Figure S2 Box‐and‐whiskers plots showing pre‐ and post‐cerclage cervical length according to pregnancy history and race. Boxes show median and interquartile range and whiskers are range. [file UOG-66-210-s001.png]

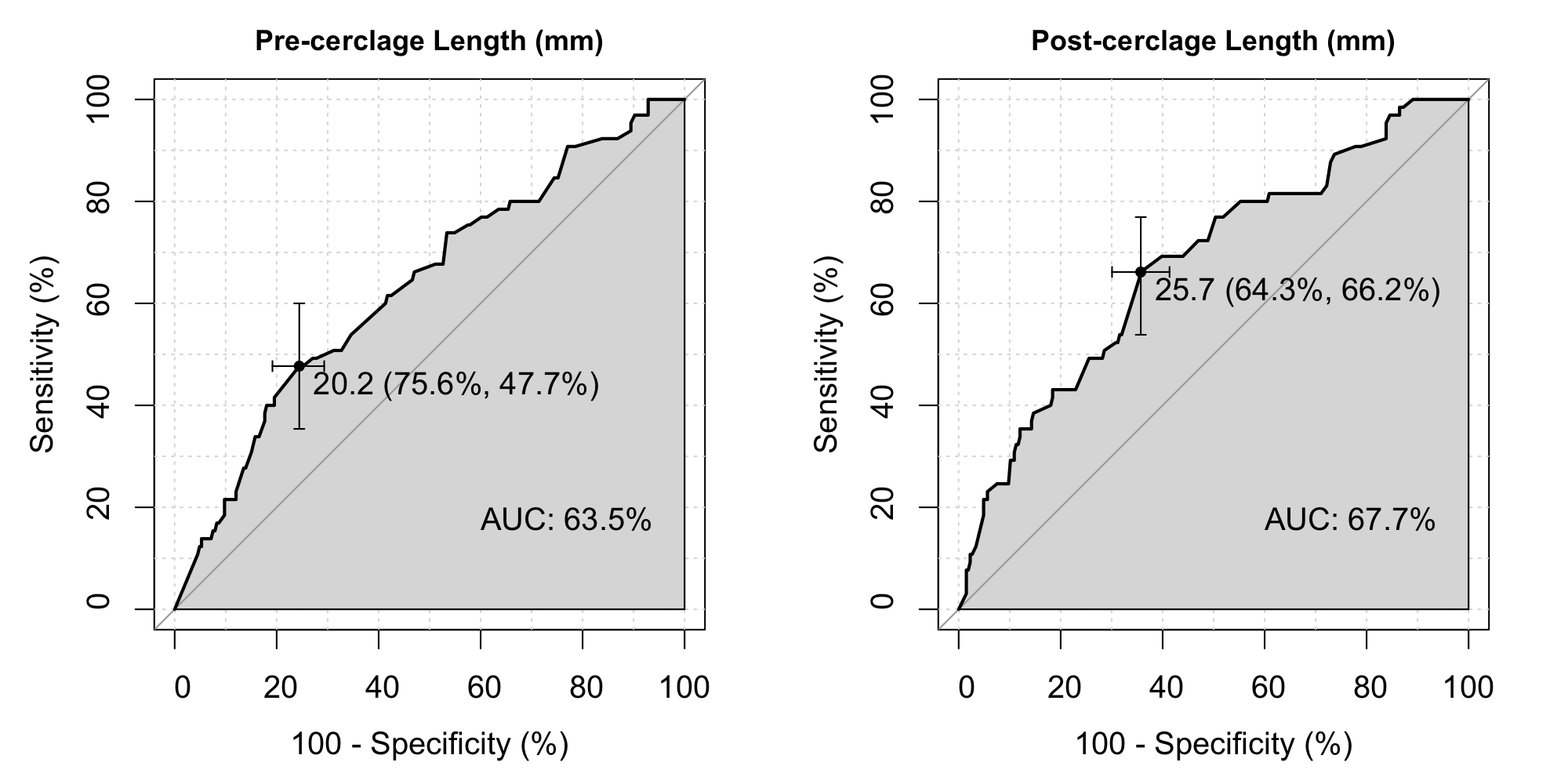

Supplement: Supplementary file 3 — Figure S3 Optimal threshold for pre‐ and post‐cerclage cervical length. [file UOG-66-210-s003.png]
