# Supplementary material for: Predictive value of cervical length for spontaneous preterm birth in women with cervical cerclage
Source: Ultrasound Obstet Gynecol. 2025 Jul 9;66(2):210–6. doi: 10.1002/uog.29281 (PMC12317304; doi:10.1002/uog.29281)
Supplement: Supplementary file 4 — Table S1 Pre‐ and post‐cerclage cervical length (CL) across risk factor groups Table S2 Logistic regression analysis to determine contribution of pre‐ and post‐cerclage length (CL) and gestational age at cerclage on delivery outcome Table S3 Logistic regression analysis to determine contribution of only pre‐ and post‐cerclage cervical length (CL) on delivery outcome [file UOG-66-210-s004.docx]

**Table S1** Pre- and post-cerclage cervical length (CL) across risk factor groups

| Parameter | | Pre-cerclage CL (mm) | Post-cerclage CL (mm) |
| --- | --- | --- | --- |
| Previous Cervical Surgery | |  |  |
| No | 27.33 (20.00–34.33) | 28.33 (21.00–35.00) |  |
| Yes | 23.50 (20.00 - 25.50) | 27.00 (22.00 - 31.75) |  |
| Pregnancy History | |  |  |
| Nulliparous | 20.00 (9.50 - 24.00) | 25.00 (17.75 - 28.50) |  |
| Nulliparous with previous MTL | 26.50 (19.75 - 34.25) | 25.00 (20.83 - 34.08) |  |
| Parous with previous MTL | 31.33 (24.33 - 35.50) | 31.67 (24.00 - 36.00) |  |
| Parous with previous iPTB | 21.10 (15.65 - 26.55) | 21.75 (17.12 - 26.38) |  |
| Parous with previous sPTB | 28.00 (22.00 - 34.00) | 30.00 (23.08 - 34.83) |  |
| Parous with previous term delivery only | 23.00 (17.00 - 28.00) | 27.50 (21.00 - 31.67) |  |
| Race | |  |  |
| Asian | 27.00 (20.50 - 31.67) | 32.00 (23.67 - 35.17) |  |
| Black | 28.50 (21.50 - 34.50) | 30.17 (22.00 - 35.42) |  |
| Mixed | 24.50 (20.00 - 33.50) | 25.50 (21.25 - 33.58) |  |
| Other | 30.17 (23.75 - 35.00) | 29.67 (23.08 - 30.75) |  |
| White | 24.00 (18.5 - 29.67) | 25.67 (20.00 - 31.62) |  |

Data are given as median (interquartile range). iPTB, iatrogenic/indicated preterm birth; MTL, mid-trimester loss; sPTB, spontaneous preterm birth.

**Table S2** Logistic regression analysis to determine contribution of pre- and post-cerclage length (CL) and gestational age at cerclage on delivery outcome.

| Predictor | Odds Ratio | CI Lower | CI Upper | *P* |
| --- | --- | --- | --- | --- |
| Pre-cerclage CL | 0.964 | 0.936 | 0.994 | 0.018 |
| Post-cerclage CL | 0.940 | 0.910 | 0.970 | < 0.001 |
| Gestational age at cerclage | 0.989 | 0.976 | 1.003 | 0.131 |

**Table S3** Logistic regression analysis to determine contribution of only pre- and post-cerclage cervical length (CL) on delivery outcome

| Predictor | Odds Ratio | CI Lower | CI Upper | *P* |
| --- | --- | --- | --- | --- |
| Pre-cerclage CL | 0.974 | 0.949 | 1.001 | 0.058 |
| Post-cerclage CL | 0.943 | 0.914 | 0.973 | < 0.001 |
